# Imageless navigation total hip arthroplasty – an evaluation of operative time

**DOI:** 10.1051/sicotj/2018016

**Published:** 2018-05-23

**Authors:** Epaminondas Markos Valsamis, David Ricketts, Adnan Hussain, Amir-Reza Jenabzadeh

**Affiliations:** 1 Hinchingbrooke Hospital NHS Trust, Huntingdon PE29 6NT UK; 2 Brighton and Sussex University Hospitals, Brighton BN2 5BE UK

**Keywords:** Imageless navigation, Hip, Arthroplasty, Operative time

## Abstract

*Introduction*: Imageless navigation has been successfully integrated in knee arthroplasty but its effectiveness in total hip arthroplasty (THA) has been debated. It has consistently been shown that navigation adds significant time and cost to the operation. Further, the relative success of traditional hip replacements has impeded the adoption of new techniques.

*Methods*: We compared the operative time between fifty total hip replacements with and without the use of imageless navigation by a single senior surgeon in a retrospective study. We employed standard statistical tools to compare the two methods. A correlation-based analysis was used to delimit the “learned” phase of imageless navigation to make comparisons meaningful.

*Results*: Contrary to what has previously been reported, there was no significant difference between operative time in navigated, when compared to traditional operations (*p* = 0.498). Only fourteen operations were required to delimit the learning phase of this operation.

*Discussion*: This is the first study that demonstrates no added operative time when using imageless navigation in THA, achieved with an improved workflow. The results also demonstrate a very reasonable learning curve.

## Introduction

Total hip arthroplasty (THA) is becoming an increasingly popular operation as the prevalence of osteoarthritis is on the rise. Inaccurate implant positioning, particularly placement beyond the Lewinnek “safe zone” (outliers), has been implicated in poor long-term postoperative outcome, higher dislocation and revision rates [1–6]. Modern technology has developed various operative adjuncts hoping to strengthen the long-term efficacy of these procedures including imaging-based and imageless navigation systems. Although imageless systems provide less information about skeletal anatomy, their lower cost, radiation hazard, technical effort and time requirement have made them increasingly favourable when compared to image-based alternatives [7].

Imageless navigation is relatively new technology which has been shown to increase accuracy in the placement of the acetabular component [7–11], and a meta-analysis has demonstrated a significant reduction in cup outliers [12]. The Australian joint registry [13] has shown navigation reduces long term revision rates in THA. Despite these advantages, imageless navigation in hip arthroplasty has been only slowly adopted into orthopaedic practice, potential reasons being its higher cost, prolonged operative time, poor workflow and the generally satisfactory outcomes that traditional hip replacements achieve. Moreover, there is a lack of evidence to support an improvement in functional outcome in THA when using imageless navigation.

Studies [7,8,14] have demonstrated up to 58 min added operative time when using imageless navigation, though less so when compared to CT-navigation. The wide range of reported increases in operative time when using imageless navigation could be the result of variation in operative speed between surgeons as well as variation in technique. Furthermore, although navigation can be used exclusively for acetabular component positioning, other surgeons may also use it to place the femoral stem and measure leg length and offset which would inadvertently increase total operative time and make comparisons unfair.

Previous studies [8,9,15] concluding on operative time and the learning curve for imageless navigation THA have not proposed a rigorous method that delimits learning from expert phases and therefore the definitions between inexperienced and experienced surgeons (in using navigation) are somewhat vague. Indeed, in evaluating and comparing any new method researchers should carefully identify the onset of expert performance in any statistical analysis to make comparisons fair. To this end we are utilising a novel two line learning curve model which allows learning to be delimited from the learned phase.

In this study, we compared the operative times in THA with and without the use of imageless navigation performed by the same surgeon. A key feature of this study is that we delineated the learning curve for navigation in terms of the operative time and determined the point at which the surgeon had, for all intents and purposes, learned the procedure. This allowed us to restrict the comparison of the two methods to the expert stages of the methods making the comparison valid and meaningful.

## Materials and methods

All operations were undertaken by the consultant surgeon (ARJ) who was proficient in traditional THA and had already undertaken 130 traditional hip replacements as a consultant. He had prior experience in the use of navigation for total knee replacements.

We analysed the surgeon’s first fifty consecutive imageless navigation-assisted THAs and determined the number of operations required to delimit the learning stage; subsequent operations were defined as “learned”.

We additionally reviewed the latest consecutive traditional THA operations by the same surgeon and compared the operative time to his “learned” navigated operations.

All operations studied were primary hip replacements. No revisions were included.

Operative records were used to source information regarding operative time, age, gender, BMI and ASA grade. We defined operative time as knife-to-skin until wound closure.

Table 1 shows the patient demographics.

**Table 1:** Patient demographics. Percentages and ranges are shown for categorical and continuous data respectively in brackets.

Factor	All (*n* = 72)	Traditional (*n* = 36)	Navigated (*n* = 36)	*p*-value
Age (years)	71.4 (54.5–86.3)	70.2 (54.5–86.3)	72.6 (60.5–85.8)	0.179
Gender	–	–	–	0.213
Male (%)	24 (36.1)	9 (25.0)	15 (41.7)	–
Female (%)	48 (63.9)	27 (75)	21 (58.3)	–
ASA	2 (1.00–4.00)	2 (1.00–3.00)	2 (1.00–4.00)	0.451
BMI	29.9 (19.0–42.0)	30.0 (19.0–42.0)	29.2 (22.0–35.0)	0.488

### Surgical technique

The patient is placed in the lateral decubitus position. They have a standard posterior support and an anterior abdominal support placed between the anterior superior iliac spine and the pubic symphysis pubis. This allows palpation of the bony point to register the Anterior Pelvic Plane. The Aesculap Orthopilot THA 1.0 Navigated surgical technique was used with a mobile tracker fixed to the iliac crest. Pelvic orientation was defaulted to the Anterior Pelvic Plane, however in high BMI patients where the ASIS is not palpable the Acetabular Centre Axis was utilised. A cup first technique with a posterior approach is utilised as per the surgical technique manual for THA Universal 1.0. The following implants were used: Aesculap plasmafit cup, Excia T stem, polyethylene insert with either a ceramic or cobalt- chromium head depending on patient age.

Apart from the use of navigation, the surgical approach was unchanged between navigated and traditional operations.

### Statistical analysis

Analysis of the data was undertaken using XLSTAT add-in software [16] on Microsoft Excel as well as SPSS statistical software [17]. *T*-tests were used to compare continuous, normally distributed data (age, BMI, ASA) whereas chi-squared tests were used to compare categorical data (gender). A *p*-value less than 0.05 was considered statistically significant.

### Modelling the learning curve

Rather than attempting to devise a model that describes a general learning scenario, we applied a simple method merely to delimit the learning phase from learned phase in the time series of operative times. This modelling technique was developed and adapted by one of the authors (EMV) [18].

Assuming that the first *k* points in a series of operations belong to the learning phase simply represented by a straight line of the form *y = mx + c* while the remaining points belong to a plateau *y = j*, we aimed to find *k* that minimises a simple convex function of the deviations of the correlation coefficients of the two phases from their ideal values and used this as the ordinal value that delimits learning from learned. This offers a simple and computationally effective method of identifying the ordinal value in the time series which separates learning from learned.

More specifically we considered points (*i, x_i_*) *i *= (1–*n*), where *i* is the ordinal value in a series and *x_i_* is the operative time of the *i*th operation. An optimal way of fitting these data to a set of two straight lines (a learning line *y = m.i + c* and a plateau *y = j*) is to choose *p* as the value of *i* that minimises *s*(*p*) where:

$$s(p)={\left[f(p)+1\right]}^{2}+{\left[g(p)\right]}^{2}\text{.}$$

*f*(*p*) is the correlation coefficient for the first *p* data points and *g*(*p*) the correlation coefficient of the remaining *n–p* data points.

The rationale behind this method is that an ideal learning phase would produce a correlation coefficient of −1 and an ideal plateau would give a correlation coefficient equal to zero. Any deviation from these ideal values contributes to an increase in *s*(*p*) and by finding the point *p* which minimizes *s*(*p*) we conjointly find a “best” compromise between learning and learned.

## Results

### Operative time

**Figure 1:**
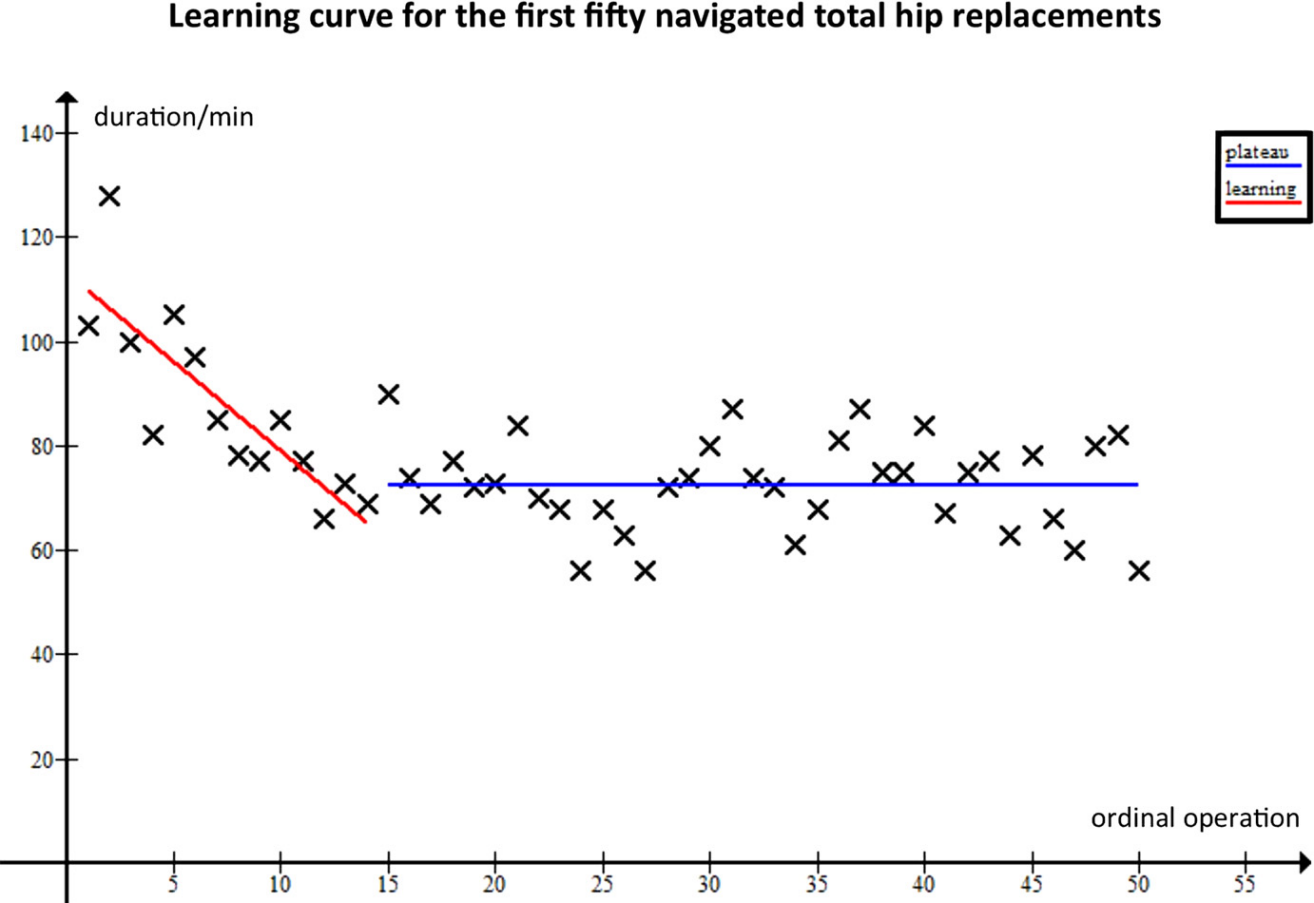
Operative time for the first fifty navigated THAs. Double straight line model superimposed.

**Figure 2:**
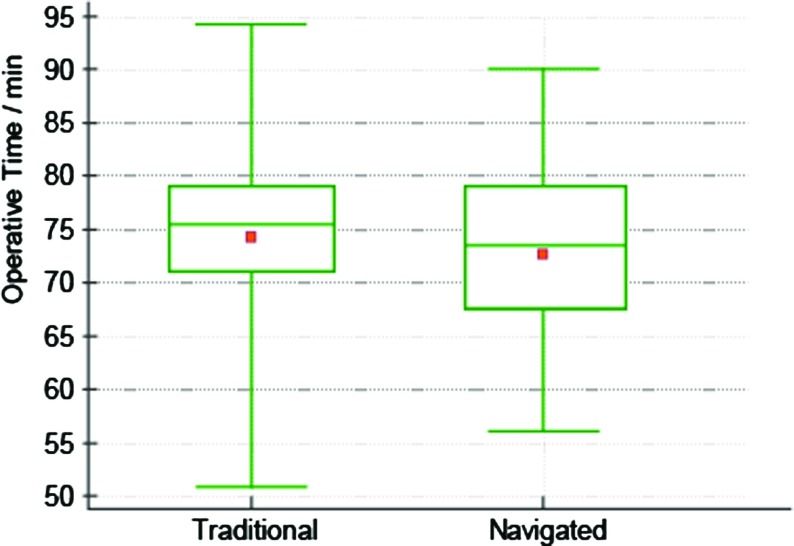
Comparison of operative time between imageless navigation and traditional operations. The means are not significantly different, *p* = 0.498.

Fourteen navigated operations were required to reach the learning plateau for navigated THAs (Figure 1). We hence compared operative time between the 36 learned navigated operations and the 36 latest traditional operations. There was no significant difference between the operative time for learned navigated (*n* = 36, mean 72.6 min) and traditional operations (*n* = 36, mean 74.2 min): *t*-test, *p* = 0.498 (Figure 2).

### Age, BMI and ASA grade

Age, BMI and ASA grade did not correlate strongly with operative time, and *p* values were not significant (Table 2).

**Table 2:** Univariate statistics. Correlation between age, BMI and ASA grade with operative time. *r* is the correlation coefficient.

Factor	Correlation
Age with operative time	*r* = 0.193, *p* = 0.105
ASA with operative time	*r* = 0.205, *p* = 0.084
BMI with operative time	*r* = −0.080, *p* = 0.506

## Discussion

In this study, we primarily set out to compare the operative time between navigated and traditional operations. To do this we delimited the learning phase by considering the learning curve for navigated THAs by a single consultant surgeon to allow meaningful comparison between “learned” operations. Our analysis shows that fourteen operations were required for the surgeon to reach a plateau in terms of operative time (72.6 min).

We did not find a significantly different operative time between navigated and traditional THAs. Although navigation improved efficiency by avoiding multiple sequential acetabular reamings and repeated trials in fitting stems to recreate offset and leg length, our result contrasts with those of other studies [7–10,14] which have suggested that navigation adds between 4.5 and 58 min to operative time. This may be explained by the choice of navigation software and workflow, as well as operative technique. The presence of a trained operator for the computer in all our operations certainly saved time. Our surgeon’s average operative times (72.6 min navigated, 74.2 min traditional) were shorter than those in all the other studies (85–124 min navigated, 77–111 min traditional) [7–10,14] and this may point to the definition of operative time as well as variability between surgeons.

The need to delimit and hence remove operations that belong to the learning phase lead us to establish a simple learning model which offers a simple way to separate learning from learned. The method uses a dual correlation analysis that determines the point in the operative series at which learned performance has been reached. This can also be confirmed on graphical inspection. Of note, our surgeon had prior experience in navigation, having used it in total knee arthroplasty. This may mean that his learning phase was shortened, and as such a greater number of operations may be required to achieve learning in a navigation-naïve surgeon. Nonetheless the purpose of the study was not to investigate the length of training for this particular operation as indeed different surgeons would likely exhibit different learning curves. Our results do, however, demonstrate that the learning phase for navigated THAs is reasonably short.

This study had a few limitations. Firstly, this was a retrospective study, and a well-designed prospective randomised control trial will be required to reliably determine the difference in operative time, and to compare between these two arthroplasty techniques. Second, radiographic analysis of acetabular cup placement such as anteversion and inclination was not possible due to the lack of standardisation in postoperative radiographs. Finally, we did not investigate functional outcome in this study.

## Conclusion

The goal of this study was to compare imageless navigation THAs with traditional operations. We demonstrated that there was no significant difference in operative time between these two techniques. This result contradicts previous research and could be due to optimised workflow, choice of software and theatre personnel familiar with navigation. This strengthens the argument for imageless navigation THA, given its other benefits which include more accurate cup positioning and reduced revision rates.

At the same time a mathematically novel approach to modelling the learning curve was used to determine the number of operations required for a surgeon to master the procedure. This model has broad application where the learning phase of a procedure must be delimited.

Our study suggests that imageless navigation for THA is a favourable adjunct to modern orthopaedic practice and the learning curve may not be as protracted as was previously thought. Further research is required, particularly in the form of prospective studies, to identify the individual contribution of specific workflow modifications in decreasing navigated operative time. More functional outcomes of imageless navigation THA patients also need to be studied.

## Conflict of interest

The authors declare that they have no conflict of interest in relation to this article.
